# Comparative metabolic fingerprinting of *Gentiana rhodantha* from different geographical origins using LC-UV-MS/MS and multivariate statistical analysis

**DOI:** 10.1186/s12858-015-0038-5

**Published:** 2015-03-28

**Authors:** Yu Pan, Ji Zhang, Tao Shen, Yan-Li Zhao, Yuan-Zhong Wang, Wan-Yi Li

**Affiliations:** Institute of Medicinal Plants, Yunnan Academy of Agricultural Sciences, 2238, Beijing Road, Panlong District, Kunming, 650200 China; College of Traditional Chinese Medicine, Yunnan University of Traditional Chinese Medicine, Kunming, 650500 China; College of Resources and Environment, Yuxi Normal University, Yuxi, 653100 China

**Keywords:** *Gentiana rhodantha*, Chemical fingerprint, LC-UV-MS/MS, Geographical origins, Characteristic compounds

## Abstract

**Backgrounds:**

*Gentiana rhodantha*, a rich source of iridoids and polyphenols, is a traditional ethnomedicine widely used in China. Metabolic fingerprinting based on a LC-UV-MS/MS method was applied to explore the chemical markers for discrimination of *G. rhodantha* from different geographical origins.

**Results:**

Targeted compounds were separated on a Shim-pack XR-ODS III (150 × 2.0 mm, 2.2 μm), with a mobile phase consisted of acetonitrile and 0.1% formic acid in water, under gradient elution. In quantitative analysis, all of the calibration curves showed good linear regression (R^2^ < less than 0.9991) within the tested ranges, and accuracy ranged from 97.8% to 104.2% and the %RSD of precision (less than 3%) were all within the required limits. The most abundant mangiferin (82.21 mg/g) found in sample from Zunyi, Guizhou province. Furthermore, 64 samples according to their geographical origins, could be classified by partial least-squares discriminate analysis (PLS-DA) and nine compounds including two new compounds identified by mass spectrometry could be regarded as characteristic compounds for discriminating samples from different geographical origins.

**Conclusions:**

The developed method appears to be a useful tool for analysis of *G. rhodantha*, which could provide potential indicators for differentiation of different geographical origins.

**Electronic supplementary material:**

The online version of this article (doi:10.1186/s12858-015-0038-5) contains supplementary material, which is available to authorized users.

## Backgrounds

Metabolic fingerprinting based on modern separation science was an effective tool for analyzing bio-samples with complex chemical information [1,2]. Currently, it showed strong potential for analysis of food and medicine as well as monitoring metabolic variation under different surrounding conditions when combined with multivariate statistical analysis [3-7]. Herbal medicines utilized as folk medicines since the ancient times are increasingly rising worldwide attention owing to its effect for maintaining health. The therapeutic effects of herbal medicines, to a large extend, were derived from synergistic effect of its metabolites. However, metabolites in herbal medicines vary with the geographical origins, which may have influence on the quality and effectiveness of herbal medicines [8-15].

*Gentiana rhodantha* Franch. ex Hemsl, Gentianaceae, is mainly distributed in southwest China [8]. The aerial parts of *G. rhodantha*, a traditional ethnomedicine, is wildly used by Tibetan and Miao nationality for treatment of hepatitis, jaundice, phthisis, dysentery, etc. [16]. In 2012, the whole plant of *G. rhodantha* was officially documented in enlarged edition of Chinese Pharmacopoeia 2010 ed. and named Entianae Rhodanthae Herba [15]. Several phytochemical and bioactivity researches demonstrated that *G. rhodantha* is a rich source of iridoids and polyphenols and showed anti-inflammatory, hepatoprotective, antimicrobial activities etc. [17-21]. Moreover, mangiferin was confirmed as the characteristic compound in *G. rhodantha* [15].

Several chromatographic methods including TLC, HPLC and UPLC-MS were developed for determination of mangiferin in *G. rhodantha* [15,22,23]*.* To the best of our knowledge no report is available on discrimination of *G. rhodantha* with different source by metabolic fingerprinting combined with multivariate analysis.

In present study, metabolic fingerprinting based on LC-UV-MS/MS method was developed for simultaneous determination of five compounds and evaluating metabolic similarities and differences in 64 batches of *G. rhodantha* from different geographical origins. Similarly analysis was applied for measuring correlations between samples from different sites. Then, principal component analysis (PCA) was used to find the resemblance and pre-classify these samples. According to PCA results, partial least squares discriminant analysis (PLS-DA) was designed for discrimination of selected samples and determined the characteristic compounds, which could provide potential indicators of *G. rhodantha* origins.

## Results and discussion

### Optimization of UFLC-UV-MS/MS analysis conditions

To achieve a desirable resolution and separation in both chromatographic fingerprint and full scanning mode, several analytical parameters including mobile phases and elution mode, flow rate, column temperature, detection wavelength critical parameter and dwell time were optimized.

**Figure 1:**
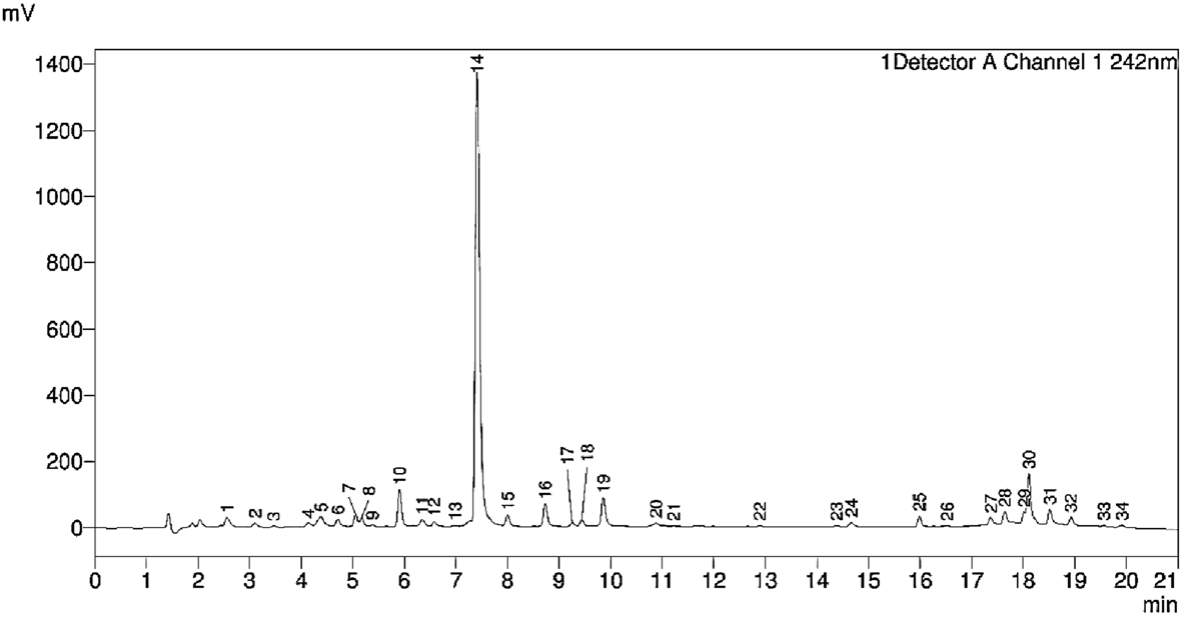
**Chromatograms at 242 nm for QC sample.**

Several types of solvent systems, including methanol–water and acetonitrile-water in various elution modes were tested to reach an optimum separation on the Shim-pack XR-ODS III (150 × 2.0 mm, 2.2 μm). A desirable separation performance was obtained in acetonitrile-water system. Then, 0.1% formic acid was added to the mobile phase to enhance resolution and eliminate peak tailing of the metabolites in fingerprints while enhancing the intensity of adducted molecular ions [M + HCOO]^−^ and protonated molecular ions [M + H]^+^ in mass spectrometer. Moreover, flow rate and column temperature was set at 0.35 min/ml and 40°C respectively, which could also improve separation efficiency. The UV wavelength was set at 242 nm, because the entire standard compounds had adequate absorptions and fingerprints also exhibited satisfactory performance (Figure 1). The optimization of mass conditions was performed in both positive and negative ionization mode. The mass range in full scanning mode was set as follow: 2–17 min at *m/z* 110–900 Da, 17–22 min at *m/z* 900–1800 Da. The critical parameter (CE) in product ions scanning mode was optimized for improving the signal of product ions according to different precursor ion. The MRM settings were auto optimized.

### Method validation and quantification

The developed method for determination of the five compounds was validated in terms of linearity, repeatability, stability, precision and accuracy. These calibration curves revealed good linear relationship and the correlation coefficients for the five compounds were greater than 0.9991. The LOD and LOQ were less than 0.007 and 0.035 μg/ml, respectively (Table 1). The validation was performed on QC sample based on retention times (Rt) and peak areas (Pa). The RSD% of repeatability and stability for Pa were less than 1.67% and 2.61%, respectively. The intra- along with inter- day %RSD of Rt were less than 1.21% and %RSD of peak areas were less than 4% (Table 2). The Recovery for all analytes in the range of low to high concentration was in the range of 97.8-104.2% (Table 3).

**Table 1:** **Linear regression data of standards**

**Analyte**	**Regression equation**	**Linearity range (** **μg/mL)**	**R** ^**2**^	**LOD (μ** **g/mL)**	**LOQ (μ** **g/mL)**
**UV detector**					
Loganic acid	y = 7230.37x + 5965.70	20-150	0.9996	0.24	1.07
Mangiferin	y = 22085.43x-36638	500-3000	0.9995	0.39	1.24
Sweroside	y = 7258.44 + 3330.77	3-60	0.9991	0.27	1.02
**MS detector**					
Gentiopicroside	y = 118727.46x + 12960.30	0.07-5	0.9997	0.014	0.049
Swertiamarin	y = 7258.44 + 3330.77	0.04-5	0.9996	0.009	0.023

**Table 2:** **Precision, repeatability and stability (%RSD) of method**

**Analytes**	**Intra-day precision RSD%**						**Inter-day**		**Repeatability**	**stability**
	**Day 1**		**Day 2**		**Day 3**		**RSD % (n = 6)**		**RSD % (n = 6)**	**RSD % (n = 6)**
	**R**_**t**_	**P**_**a**_	**R**_**t**_	**P**_**a**_	**R**_**t**_	**P**_**a**_	**R**_**t**_	**P**_**a**_	**P**_**a**_	**P**_**a**_
Loganic acid	0.63	1.49	1.03	1.57	0.98	2.51	0.58	1.67	1.33	2.36
Mangiferin	0.77	2.40	1.16	1.54	1.21	1.32	0.76	1.85	1.45	2.61
Sweroside	0.88	1.78	0.69	1.81	0.94	2.61	0.79	2.76	1.67	2.58
Swertiamarin	1.02	1.36	0.87	1.56	1.06	2.11	1.07	1.21	0.98	2.27
gentiopicroside	0.91	1.35	0.69	2.07	0.83	2.13	1.03	1.66	0.74	1.97

R_t_: retention times.

P_a_: peak areas.

**Table 3:** **Recovery study of the method (n = 3)**

**Analytes**	**Original**	**Amount**	**Measured**	**Recovery**	**RSD**
	**Amount (μ** **g/mL)**	**added (μ** **g/mL)**	**Amount (μ** **g/mL)**	**(%)**	**(%)**
Loganic acid	26.14	12.00	38.03	99.1	1.79
		24.00	50.21	101.9	2.40
		36.00	63.22	103	3.11
Swertiamarin	1.82	0.90	2.75	103.3	1.73
		1.80	3.58	97.8	1.87
		2.40	4.17	97.9	2.23
Mangiferin	601.78	300.00	906.12	101.4	1.92
		600.0	1195.88	99.0	3.07
		1200.00	1843.05	103.4	2.19
Gentiopicroside	1.24	0.60	1.83	98.3	2.25
		1.20	2.49	104.2	1.88
		2.40	3.62	99.2	1.81
	9.38	4.50	13.71	96.9	1.96
Sweroside		9.00	18.73	103.9	2.81
		18.0	27.56	101.	2.89

The five compounds were identified by comparison of their retention times and precursor/product-ion pairs obtained by MRM acquisition mode. The established method was applied for simultaneous determination of the five compounds in 64 samples of *G. rhodantha*. Among them, swertiamarin and gentiopicroside with low concentration was determined by MRM acquisition mode. Each sample was analyzed in triplicate to determine a mean content (mg/g) and standard deviation (SD). As shown in Table 4, there were significant differences in compounds contents of *G. rhodantha* from different geographical origins. Mangiferin was found to be predominant in *G. rhodantha*, ranging from 45.19 to 82.21 mg/g. The yield of mangiferin followed in the order: GZ > YM > GXL > GAS > YK > GX > YD > GAL > YW > YL > GK. In addition, the highest yield of sweroside (6.10 ± 1.50 mg/g) was found in Guangxi province.

**Table 4:** **Mean contents (mg/g) of the five compounds in** ***G. rhodantha***

**NO.**	**Loganic acid**	**Mangiferin**	**Sweroside**	Swertiamarin*	Gentiopicroside*
GAL (n = 6)	3.09 ± 0.66	52.74 ± 2.51	1.19 ± 0.36	0.023	0.012
GAS (n = 6)	2.54 ± 0.31	61.07 ± 2.18	3.26 ± 0.33	-	0.008
GX (n = 6)	2.75 ± 0.63	55.27 ± 4.87	-	0.033	-
GK (n = 6)	1.33 ± 0.29	40.74 ± 4.85	-	0.009	0.021
GZ (n = 6)	1.71 ± 0.15	82.21 ± 16.25	1.57 ± 0.24	-	0.028
YL (n = 6)	3.70 ± 0.29	45.19 ± 4.57	1.78 ± 0.12	-	0.039
YD (n = 6)	3.30 ± 0.28	53.79 ± 6.43	-	-	0.007
YM (n = 6)	3.05 ± 0.08	65.44 ± 10.1	1.06 ± 0.11		0.018
YW (n = 6)	2.33 ± 0.12	50.87 ± 8.29	-	0.023	-
YK (n = 6)	4.45 ± 0.47	59.41 ± 11.2	-	0.008	0.015
GXL (n = 4)	3.18 ± 0.30	62.60 ± 11.7	6.10 ± 1.50	-	-

-: lower than limit of quantification.

*Determined by MRM.

**Table 5:** **Marker metabolites in** ***G. rhodantha***

**Peak No.**	**Rt (min)**	**precursor ion (** ***m/z*** **)**	**CE (eV)**	**MS/MS fragment (Relative abundance %)**	**Identity**	**Ref**
4	4.2	423.30 [M-H]^−^	−24	423 (24) 261 (100) 151 (32) 125 (8)	rhodanthenones B	[20]
		425.20 [M + H] ^+^	−13	425 (18) 263 (100) 145 (19) 127 (8)		
7	5.1	375.10 [M-H]^−^	22	213 (100)	loganic acid*	
9	5.5	405.15 [M + H]^+^	−16	405 (31) 378 (12) 243 (100) 211 (63)	secoxyloganin	[19]
		449.20 [M + HCOO]^−^	21	449 (27) 403 (22) 359 (26) 241 (100) 197 (29) 179 (77) 161 (18) 144 (17) 127 (15)		
10	5.9	413.30 [M + Na]^+^; 389.20 [M-H]^−^	25	389 (21) 345 (11) 227 (22) 209 (8) 183 (23) 165 (31) 139 (34) 121 (100) 113 (48) 95 (28)	secologanoside	[19]
12	6.6	419.15 [M + HCOO]^−^	15	179 (100)	swertiamarin*	
		375.10 [M + H]^+^	−12	195 (100), 177 (63)		
14	7.4	421.25 [M-H]^−^	30	301 (100), 331 (71)	mangiferin*	
—	7.5	401.10 [M + HCOO]^−^	20	355 (23) 179 (100)	gentiopicroside*	
15	7.9	403.20 [M + HCOO]^−^	21	357 (29) 195 (32) 125 (100)	sweroside*	
20	10.9	449. 10 [M + H]^+^ 447.20 [M-H]^−^	−15	449 (13) 431 (47) 413 (65) 383 (43) 359 (22) 329 (100)	isoorientin	[21,24]
27	10.5	1075.40 [M-H]^−^; 1099.10 [M + Na]^+^	35	1075 (51), 913 (59) 555 (100) 357 (28) 197 (32) 153 (31)	unknow	[17,18]
28	10.8	1433.30 [M-H]^−^; 1452 [M+ NH_4_]^+^	32	1433 (17) 1271 (100) 1075 (43) 913 (84) 555 (100) 197 (20) 153 (17)	unknow	[17,18]
29	18.0	913 [M-H]^−^; 937.13 [M + Na]^+^	39	913 (14), 555 (55), 357 (12), 197 (100) 153 (61) 109 (18)	rhodenthoside A	[17,18]
30	18.1	1629 [M-H]^−^; 1648 [M + NH_4_]^+^	63	1629 (7) 1271 (37) 913 (93) 555 (100) 197 (29) 153 (18)	rhodenthoside B	[17,18]
31	18.5	1643 [M-H]^−^; 1667 [M + Na]^+^	55	1643 (27) 1285 (100) 1271 (33) 927 (36) 913 (53) 555 (37) 197 (29) 153 (22)	unknow	[17,18]
32	18.9	1657 [M-H]^−^; 1681 [M + Na]^+^	59	1657 (41) 1285 (32) 927 (73), 913 (31) 555 (74) 197 (33) 153 (100) 109 (29)	rhodenthoside C	[17,18]

*identification by standard compounds.

—: not detect in chromatogram.

### Similarly analysis

The similarly analysis of chromatographic fingerprint was performed by Chinese Pharmacopoeia Committee (Version 2004 A) and SPSS 20.0. The common peaks confirmed by the RSD% of all peaks’ relative retention time (<1%) were matched automatically. All fingerprints were classified into 11 groups according to their origins. Then, standard fingerprint of each group was obtained by comparing chromatograms of samples from the same origin. The correlation coefficients of fingerprint of samples from the same and different geographical origins are listed in Additional file 1: Table S1. The results showed correlation coefficients of fingerprint of samples from the same origin were greater than 95%. As shown in Additional file 2: Figure S1, samples from different origin had the same peak numbers, but peak areas and correlation coefficients were visually different in these 11 groups (Additional file 1: Table S1). For example, correlation coefficients (0.891) implied samples from Dali and Lijiang were more similar when compared with other samples. Moreover, peak area (No.20) in their fingerprints was greater than others (Additional file 2: Figure S1). These results indicated that geographical origins might have influence in metabolites accumulation.

### PCA and PLS-DA

PCA, an unsupervised classification approach, was applied for dimensions of complex data obtained from chromatographic fingerprint and discrimination of different sample. The peak areas without any pre-processing obtained from chromatographic fingerprints were subjected to PCA analysis. In Additional file 3: Figure 2S, 64 samples from 11 sites could not achieve a good classification performance. However, these samples from geographical close cities in Yunnan and Guizhou province could be clustered together, which would provide a basis for classification of these samples for further analysis.

**Figure 2:**
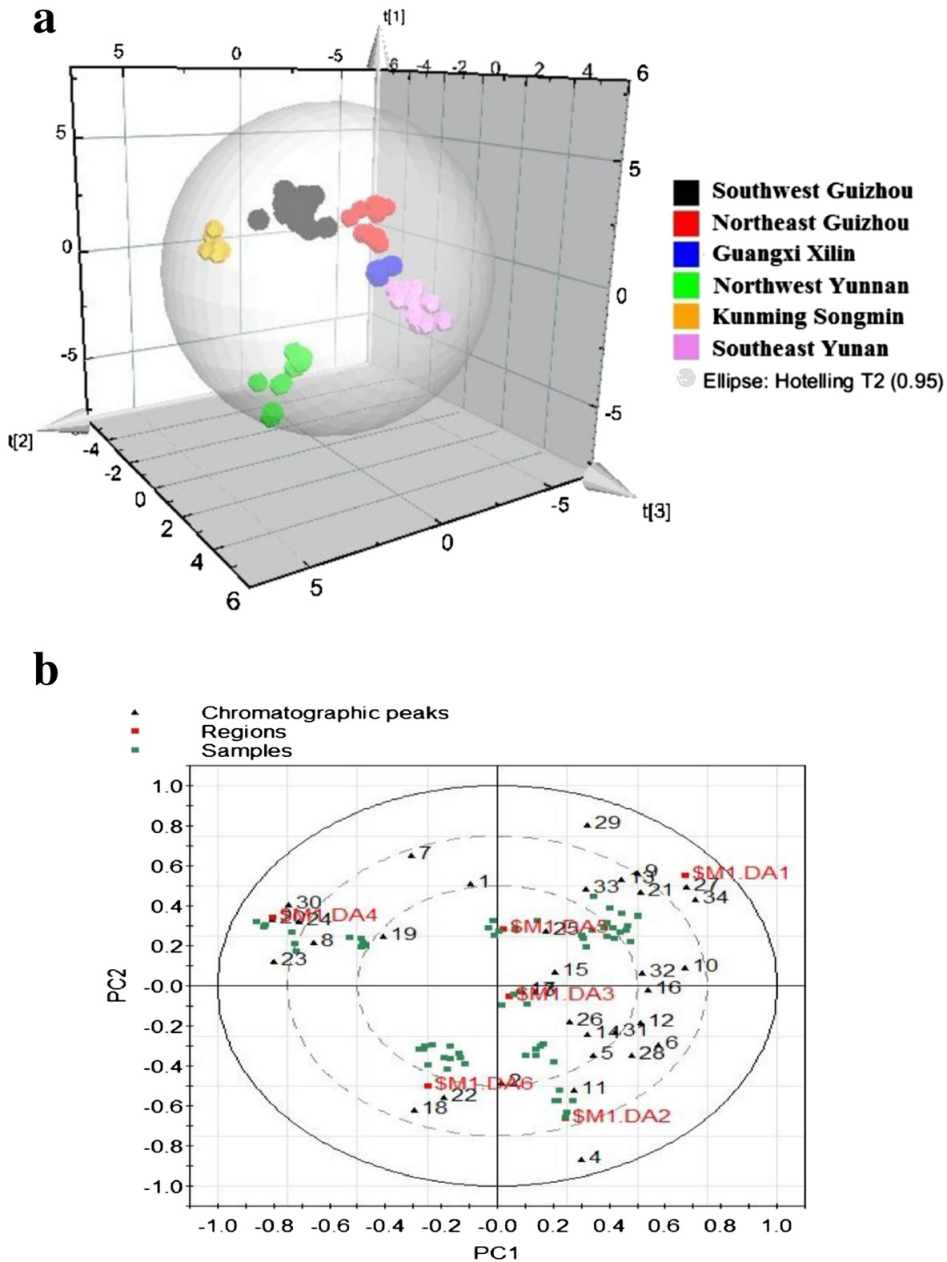
**PLS-DA 3D scores plot (a) and** **Loading Bi-plot (b) of PLS-DA model on different geographical origins of** ***G. rhodantha.*** Detailed legend of Figure 3b: (DA1: southwest Guizhou; DA2: northeast Guizhou; DA3: Xilin DA4: northwest Yunnan; DA5: Kunming; DA6: southwest).

According to PCA results, PLS-DA, a supervised classification approach, was designed for further discrimination of these samples. As shown in PLS-DA scores plot (Figure 2a), 64 samples from 6 main geographical origins (northwest, east and southeast of Yunnan, northeast and southwest of Guizhong as well as Guangxi) were well separated. Among these origins, Linxian, Guangxi province is located between southeast Yunan and Guizhou. The relative location of samples from the three regions in PLS-DA scores plot conformed to the actual geographic location. Moreover, samples from different origins in Yunnan were significant different, which might owe this difference to the complicated climatic and geographical conditions in Yunnan.

In loading-bi plot (Figure 2b), the important principal components and chemical compounds in separating the samples could be exhibited. PC1 have potent impact on discrimination of samples in Yunnan from others. Moreover, peak 34 and 27 might have more influence on discrimination of samples from southwest Guizhong; Peak 17 and 15 play important roles for the discriminating GXL samples from other places; Peak (2, 18 and 22) and Peak (20, 24 and 30) likely to have more contribution for classification of samples from northwest Yunnan. Interestingly, these results could correspond to the results of similar analysis and characteristic in their corresponding fingerprints.

### Marker metabolites screening and identification

**Figure 3:**
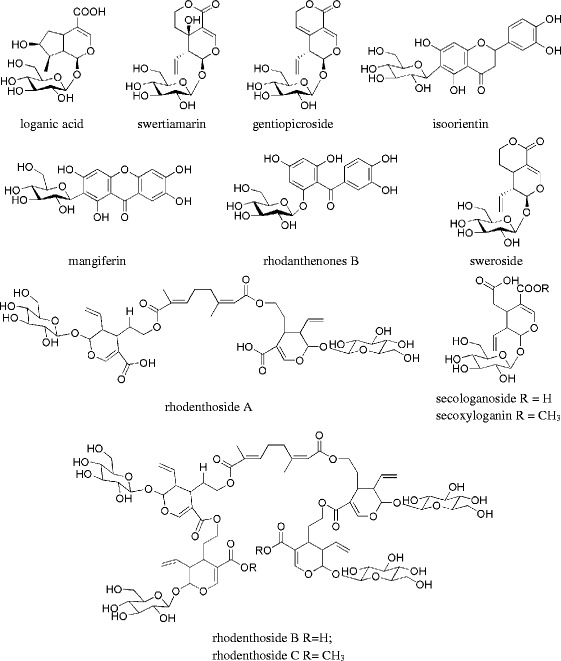
**Structures of the marker metabolites in** ***G. rhodantha*** **.**

Variable importance plot (VIP) based on PLS-DA result was used for screening the characteristic compounds according to contribution values for discrimination of different samples. In this study, peak 32, 27, 4, 30, 31, 9, 10, 29 and 20 (descending order) were likely to be considered as characteristic compounds of *G. rhodantha* in accordance with their VIP values (>1.2). These characteristic compounds were identified using LC-MS/MS operated at multiple scanning modes and literature data on the fragment ions patterns of these compounds. Marker metabolites were tentatively identified (Table 5) and their structures are displayed in Figure 3.

First of all, Peak 7, 12, 14 and 15 were identified as loganic acid, swertiamarin, mangiferin and sweroside through comparing by retention times and mass data of reference compounds.

**Figure 4:**
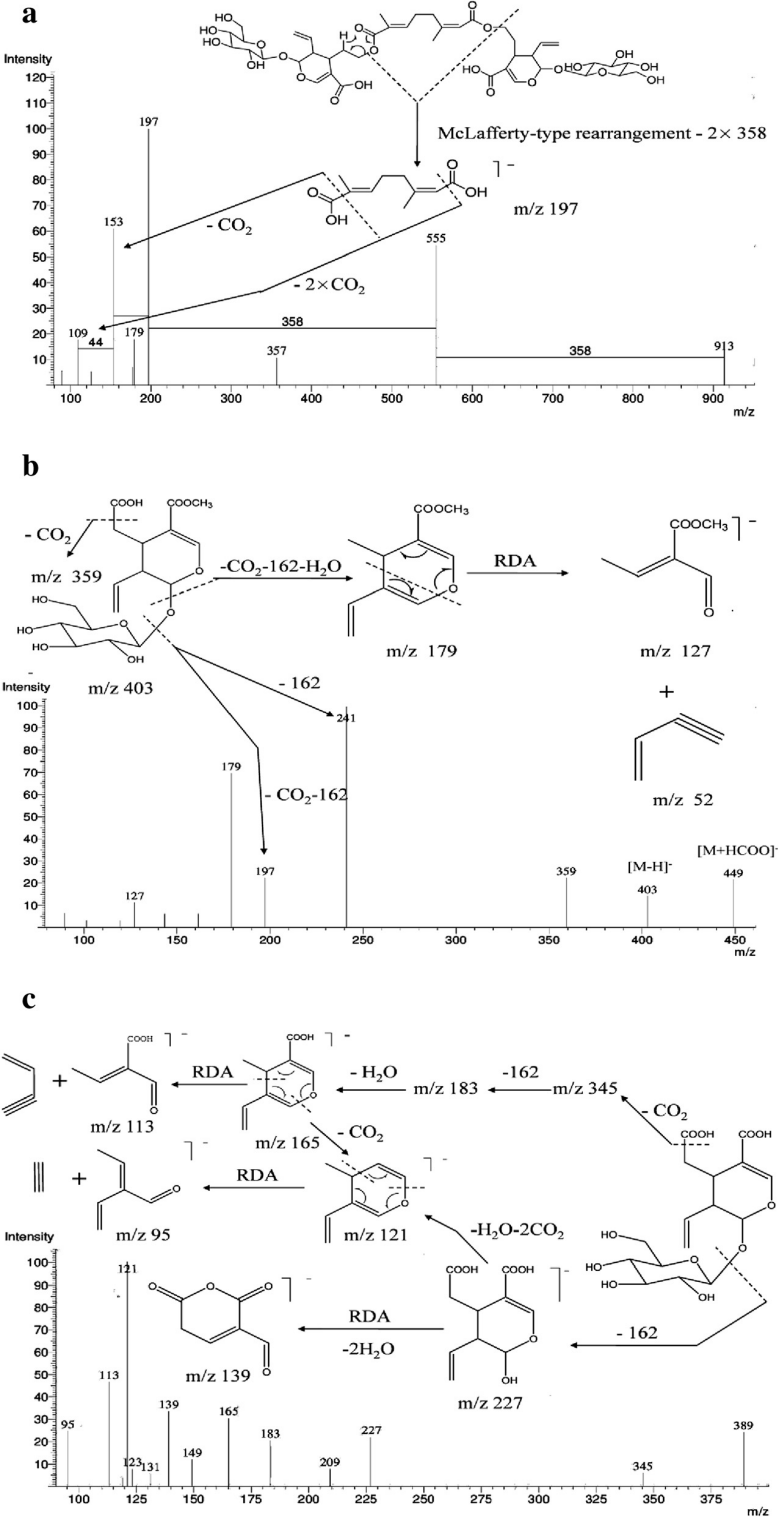
**The MS** ^**2**^ **spectra of the [M-H]** ^**−**^ **ions for (a) rhodenthoside A, (b) secoxyloganin and (c) secologanoside.**

According to the mass data analysis of peak 27–32, the major product ions and fragmentation pattern of the revealed their derivatives with the same basic skeleton. Among them, peak 29 (*m/z* 913 [M-H]^−^), 30 (*m/z* 1629 [M-H]^−^) and 32 (*m/z* 1657 [M-H]^−^) were tentatively assigned as rhodenthoside A, B and C by matching the fragmentation pattern from published works [17,18]. In negative mode, their fragment ions patterns were highly similar. As shown in Table 6 and Figure 4a, same fragmentation ions (*m/z* 555, 197, 153 and 109) were detected in their mass spectrum. Using peak 29 as an example, *m/z* 555 and 197 might be derived from *m/z* 913 through classical McLafferty-type rearrangement successively losing series of 358 (swerosidic acid moiety) while *m/z* 153 and 109 were considered as *m/z* 197 (monoterpene dicarboxylic acid moiety) successively lose series of CO_2_ (Figure 4a). Moreover, fragment ions of peak 30 (*m/z* 1271, 913, 555 and 197) were corresponded to losing four 358 moiety in succession. In fragmentation pattern of peak 32, *m/z* 1285 might be derived from losing a 372 moiety regarded as swerosidic acid methyl ester and *m/z* 927 were corresponded to [M-H-372-358]^−^. Interestingly, the same fragment ions (*m/z* 555, 197, 153) were also found in peak 27 (*m/z* 1075 [M-H]^−^), 28 (*m/z* 1433 [M-H]^−^) and 31 (*m/z* 1643 [M-H]^−^), which implied that the three compounds might be the derivatives of rhodenthosides A-C (Additional file 4: Figure S3). In peak 27, present of ions at *m/z* at 913 in negative mode (rhodenthoside A) corresponded to loss of a glucose moiety from precursor ions (*m/z* 1075), which indicated that peak 27 would tentatively assigned as glucosyl rhodenthoside A. The product ions at *m/z* 1271 and *m/z* 1075 corresponded to the eliminations of swerosidic acid and glucose moiety respectively, which implied the structure of peak 28 (*m/z* 1433 [M-H]^−^) would consider as rhodenthoside A adding a glucose and swerosidic acid. Furthermore, fragment ions of peak 31 at *m/z* 1285, 1271, 927 and 913 corresponded to the successive loss of 372 and 358 moiety, which implied the structure of peak 31 might be a product of methyl esterification of rhodenthoside B. According to mass data, peak 27, 28 and 31 were three new derivatives of rhodenthoside, whereas their structure should further confirmed by NMR.

**Table 6:** **List of samples**

**Sample NO.**	**Origin**	**Site**
GAL (1–6)	Southwest	Anlong
GAS (1–6)	Guizhou	Anshun
GX (1–6)		Xinyi
GK (1–6)	Northeast	Kaili
GZ (1–6)	Guizhou	Zunyi
YL (1–6)	Northwest	Lijiang
YD (1–6)	Yunnan	Dali
YM (1–6)	Southeast Yunnan	Mengzi
YW (1–6)		Wenshan
YK (1–6)	East Yuan	Kunmng, Songming
GXL (1–4)	Guangxi	Xinlin

Peak 9 yielded a sodium adduct ion at *m/z* 405 and a adduct ion 449 [M + HCOO]^−^ while peak 10 presents *m/z* 413 [M + Na]^+^ and 389 [M-H]^−^. In negative mode, product ions at *m/z* at 227 and 209 corresponded to losing a glucose moiety (162 Da) and H_2_O from their deprotonation ion [M-H]^−^. In addition, the common neutral loss of 44 and 52 were observed, which belong to eliminations of CO_2_ and losing a vinylethyne through retro-Diels-Alder (RDA) reaction from product ions at *m/z* at 223 and 209. According to matching the fragment ions pattern from published work [19], peak 9 and 10 were tentatively assigned as secologanoside and secoxyloganin, respectively. The fragmentation pathways of secologanoside and secoxyloganin were exhibited in Figure 4b and c.

## Conclusion

In present study, a simple and reliable method was developed for simultaneous determination of the main compounds in *G. rhodantha*. Metabolic fingerprint based on LC-UV-MS/MS was design for elucidating the change in samples from different geographical origins. The multivariate analysis demonstrated that metabolites of *G. rhodantha* were clearly dependent on geographical. Furthermore, five characteristic compounds were considered as the most important variables for distinguishing different samples according to their origins. Metabolic fingerprint based on LC-UV-MS/MS could be a useful tool for simple discrimination of *G. rhodantha* from different geographical origins when combined with multivariate analysis. The results could be consistent with the practical geological setting, which may reflect potential relationship between geographical origins and accumulation of characteristic compounds for further study.

## Methods

### Materials and reagents

**Figure 5:**
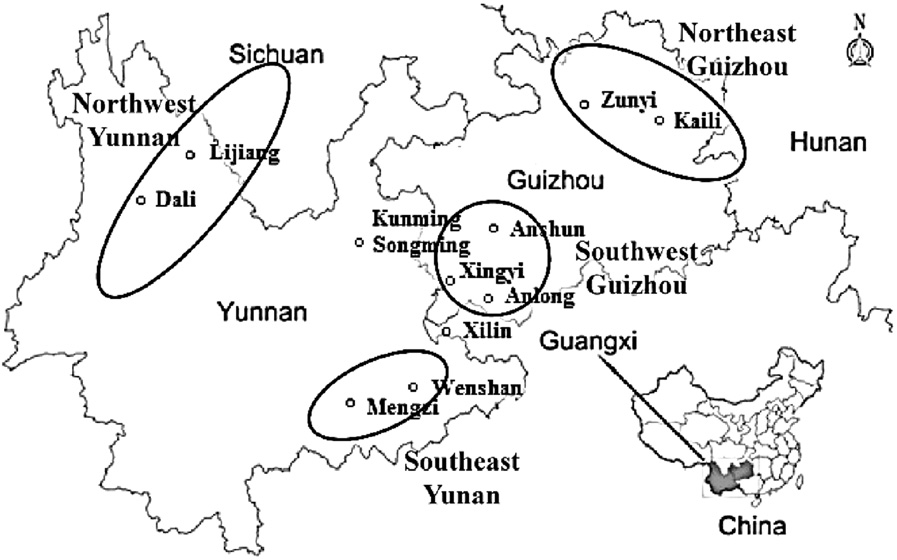
***G. rhodantha*** **collected from different geographical origins in China.**

The aerial parts of *G. rhodantha* were collected from southwest China including 11 sites (Figure 5) in January, 2014. In total of 64 samples were authenticated by Professor Hang Jin (Institute of Medicinal Plants, Yunnan Academy of Agricultural Sciences). Fresh plants were dried at 60°C and then ground into fine powder around 60 meshes. The information of samples is listed in Table 6. No specific permits were required for the described field studies, as no endangered or protected species were sampled, and the localities where the samples came from are not protected in any way.

The LC grade solvents (acetonitrile and formic acid) purchased from Fisher Scientific and Dikmapure (USA), respectively were used for LC analysis. The pure water for extraction and LC analysis was purified by a Milli-Q system from Millipore (USA). All chemicals used for extraction were analytical grade. The standard compounds (gentiopicroside, swertiamarin, loganic acid, sweroside and mangiferin) were provided by Chinese National Institute for the Control of Pharmaceutical and Biological Products (Beijing, China).

### LC-UV-MS/MS condition

Ultra-fast liquid chromatography tandem mass spectrometry (Shimadzu, LCMS-8030, Japan) was equipped with auto-sample, binary gradient pumps, ultraviolet detector, electrospray ionization interface and triple quadrupole mass spectrometer detector. Chromatographic separation was performed on Shim-pack XR-ODS III (150 × 2.0 mm, 2.2 μm). The mobile phase was consisted of 0.1% formic acid in water (A): acetonitrile (B) using gradient elution with the flow rate 0.35 ml/min as follow: 93%-90% A at 0–1.62 min, 90%-74% A at 1.62-14.72 min, 74%-20% A at 14.72-22.0 min. The column temperature was kept 40°C and the injection volume was 3 μl. The detection wavelength was set at 242 nm for fingerprinting and quantitative analysis. The mass spectrometer parameters set as follows: nebulizing gas and drying gas were nitrogen at a flow rate of 3.0 and 15.0 L/min, respectively; the interface voltage was set to 4.5 kV; desolvation line (DL) temperature was 250°C and the heat block temperature was 400°C. Data were collected in negative and positive ion modes and full scan within the range of *m/z* 100–1800 Da. Multiple reaction monitoring (MRM) acquisition mode and products ion scanning was used for quantification purposes of chemical constituents with low content and qualitative analysis, respectively.

### Preparation of standard solutions and test sample

For quantitative analysis, 2.5 mg of mangiferin was dissolved in 10 ml of 50% methanol. Other standard solutions (0.5 mg/ml) were prepared individually in methanol. All stocks solutions were stored at 4°C before LC-MS/MS analysis. Each standard solution was diluted to appropriate concentration range for establishment of calibration curves which were used for quantification by LC-UV-MS/MS detection.

An accurately weighed *G. rhodantha* powder (0.05 g) was extracted with 5 ml of 60% methanol by ultrasonic extraction at room temperature for 30 min and the extracts’ solution were filtered through a paper filter. Then, the filtrates were stored at 4°C and filtered through a 0.22 μm membrane filter before injection into the LC system for analysis.

### Method validation

In order to assure the validity of this developed method, quality control (QC) samples obtained by pooling the same amount of samples from each site were selected for method validation. The calibration curves of each standard plotted with six different concentrations were calculated using peak area (y) and concentration (x, μg/ml). The limits of detection (LOD) and quantification (LOQ), S/N (signal-to-noise ratio) of 3 and 10, were determined by serial dilution of standard solution using the described conditions. Linearity data, LODs and LOQs are summarized in Table 1.

Precision was evaluated by intra- and inter-day variation which determined by analyzing mixed standard solutions with known concentration six times within a day and on three consecutive days in triplicate, respectively. To investigate the repeatability, six independently samples were analysed by the described chromatographic condition. The stability was determined by analyzing QC sample extract six times (0, 4, 8, 12, 16, 20 and 24 h) during a single day. Accuracy was evaluated by recovery test which was performed by adding three different amounts (low, medium and high concentration) of each standard solution to the crude sample. The recovery rate was calculated by the equation as follow:

$$ \%\mathrm{R}=\left[\left(\mathrm{measured}\kern0.5em \mathrm{amount}\kern0.5em \hbox{-} \kern0.5em \mathrm{original}\kern0.5em \mathrm{amount}\right)\kern0.5em /\kern0.5em \mathrm{amount}\kern0.5em \mathrm{added}\right]\kern0.5em \times \kern0.5em 100\% $$

### Data preprocessing

The Similarity Evaluation System for Chromatographic Fingerprint of Tradition Chinese Medicine software developed by the Chinese Pharmacopoeia Committee (Version 2004 A) was used for fingerprinting analysis and calculating the similarity values in entire chromatographic fingerprint among samples. Additionally, the correlation coefficient of samples from each site was carried out in IBM SPSS 20.0 for Windows (IBM, USA). The chemometric techniques of PCA and PLS-DA were performed by software SIMCA-P^+^10.0 (Umetrics AB, Sweden).

## Additional files

Additional file 1: Table S1. Similarity comparison of fingerprints of samples from different sites. Additional file 2: Figure S1. Sample additional file title Metabolic fingerprints of *G. rhodantha* from different geographical origins. Additional file 3: Figure S2. PCA scores plot on different sites of *G. rhodantha*. Additional file 4: Figure S3. The MS^2^ spectrum of the peak 27, 28 and 31.

## Abbreviations

G: Gentiana
LC-UV-MS/MS: Liquid chromatography- ultraviolet detector- tandem mass spectrometry
MRM: Multiple reaction monitoring
NMR: Nuclear magnetic resonance
PCA: Principal component analysis
PLS-DA: Partial least squares discriminant analysis
RDA: Retro-Diels-Alder
VIP: Variable importance plot
