# Supplementary material for: Comparative metabolic fingerprinting of Gentiana rhodantha from different geographical origins using LC-UV-MS/MS and multivariate statistical analysis
Source: BMC Biochem. 2015 Mar 28;16:9. doi: 10.1186/s12858-015-0038-5 (PMC4390080; doi:10.1186/s12858-015-0038-5)
Supplement: Additional file 1: Table S1. — Similarity comparison of fingerprints of samples from different sites. [file 12858_2015_38_MOESM1_ESM.doc]

| **Table S1** **Similarity comparison of fingerprints of samples from different sites** | | | | | | | | | | | |
| --- | --- | --- | --- | --- | --- | --- | --- | --- | --- | --- | --- |
| NO. | GAL (n=6) | GAS (n=6) | GK (n=6) | GXL (n=6) | GX (n=6) | GZ (n=6) | YD (n=6) | YK (n=6) | YL (n=6) | YM (n=6) | YW (n=6) |
| GAL | 0.985±0.12^a^ | 0.849^b^ | 0.701^b^ | 0.722^b^ | 0.928^b^ | 0.543^b^ | 0.484^b^ | 0.674^b^ | 0.388^*^ | 0.498^b^ | 0.580^b^ |
| GAS |  | 0.976±0.21^a^ | 0.749^b^ | 0.747^b^ | 0.899^b^ | 0.595^b^ | 0.456^b^ | 0.735^b^ | 0.414^*^ | 0.586^b^ | 0.611^b^ |
| GK |  |  | 0.981±0.09^a^ | 0.665^b^ | 0.675^b^ | 0.750^b^ | 0.533^b^ | 0.703^b^ | 0.516^b^ | 0.707^b^ | 0.719^b^ |
| GXL |  |  |  | 0.973±0.14^a^ | 0.674^b^ | 0.734^b^ | 0.609^b^ | 0.635^b^ | 0.637^b^ | 0.653^b^ | 0.701^b^ |
| GX |  |  |  |  | 0.958±0.21^a^ | 0.521^b^ | 0.530^b^ | 0.689^b^ | 0.390^*^ | 0.496^b^ | 0.538^b^ |
| GZ |  |  |  |  |  | 0.975±0.20^a^ | 0.501^b^ | 0.688^b^ | 0.493^b^ | 0.713^b^ | 0.764^b^ |
| YD |  |  |  |  |  |  | 0.957±0.18^a^ | 0.566^b^ | 0.891^b^ | 0.616^b^ | 0.614^b^ |
| YK |  |  |  |  |  |  |  | 0.968±0.28^a^ | 0.597^b^ | 0.492^b^ | 0.572^b^ |
| YL |  |  |  |  |  |  |  |  | 0.984±0.09^a^ | 0.572^b^ | 0.554^b^ |
| YM |  |  |  |  |  |  |  |  |  | 0.971±0.13^a^ | 0.944^b^ |
| YW |  |  |  |  |  |  |  |  |  |  | 0.958±0.25^a^ |
| ^a^ : The similarity of each chromatogram to themselves simulative reference chromatogram, mean±S.D.  ^b^: The similarity between simulative reference chromatogram. | | | | | | | | | | | |
