# Supplementary figures and images for: Comparative metabolic fingerprinting of Gentiana rhodantha from different geographical origins using LC-UV-MS/MS and multivariate statistical analysis
Source: BMC Biochem. 2015 Mar 28;16:9. doi: 10.1186/s12858-015-0038-5 (PMC4390080; doi:10.1186/s12858-015-0038-5)

**Fig. S1**


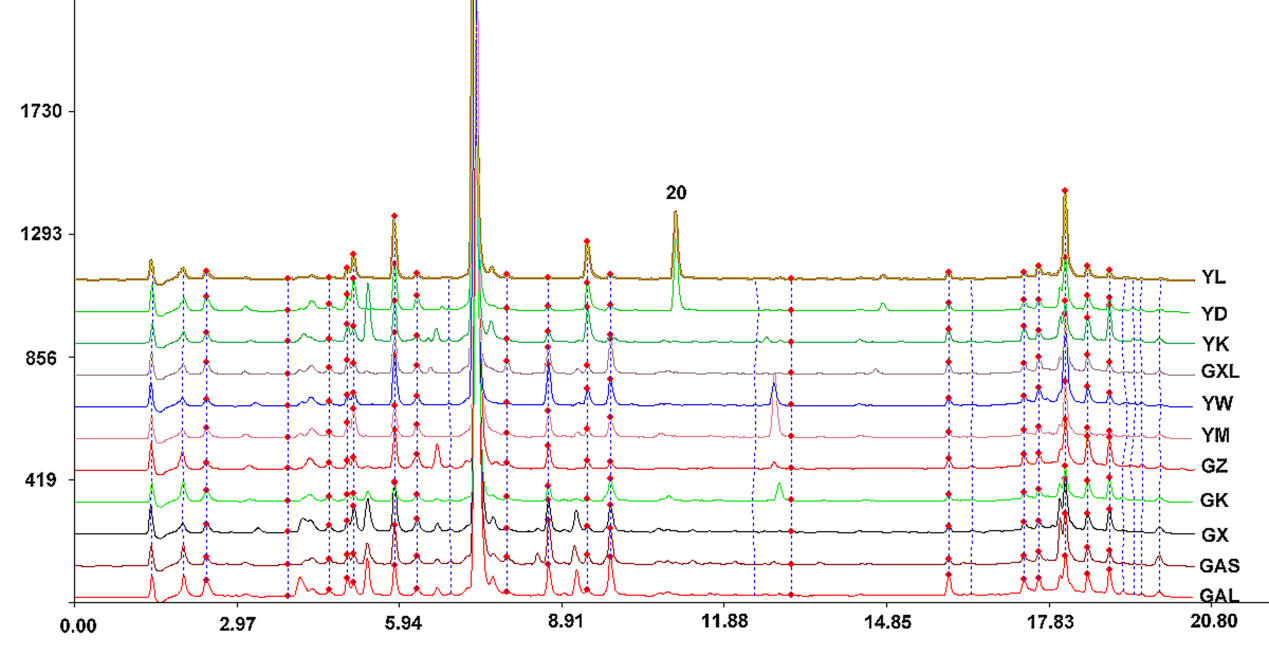

Supplement: Additional file 2: Figure S1. — Sample additional file title Metabolic fingerprints of G. rhodantha from different geographical origins. [file 12858_2015_38_MOESM2_ESM.doc]

**Fig. S2**


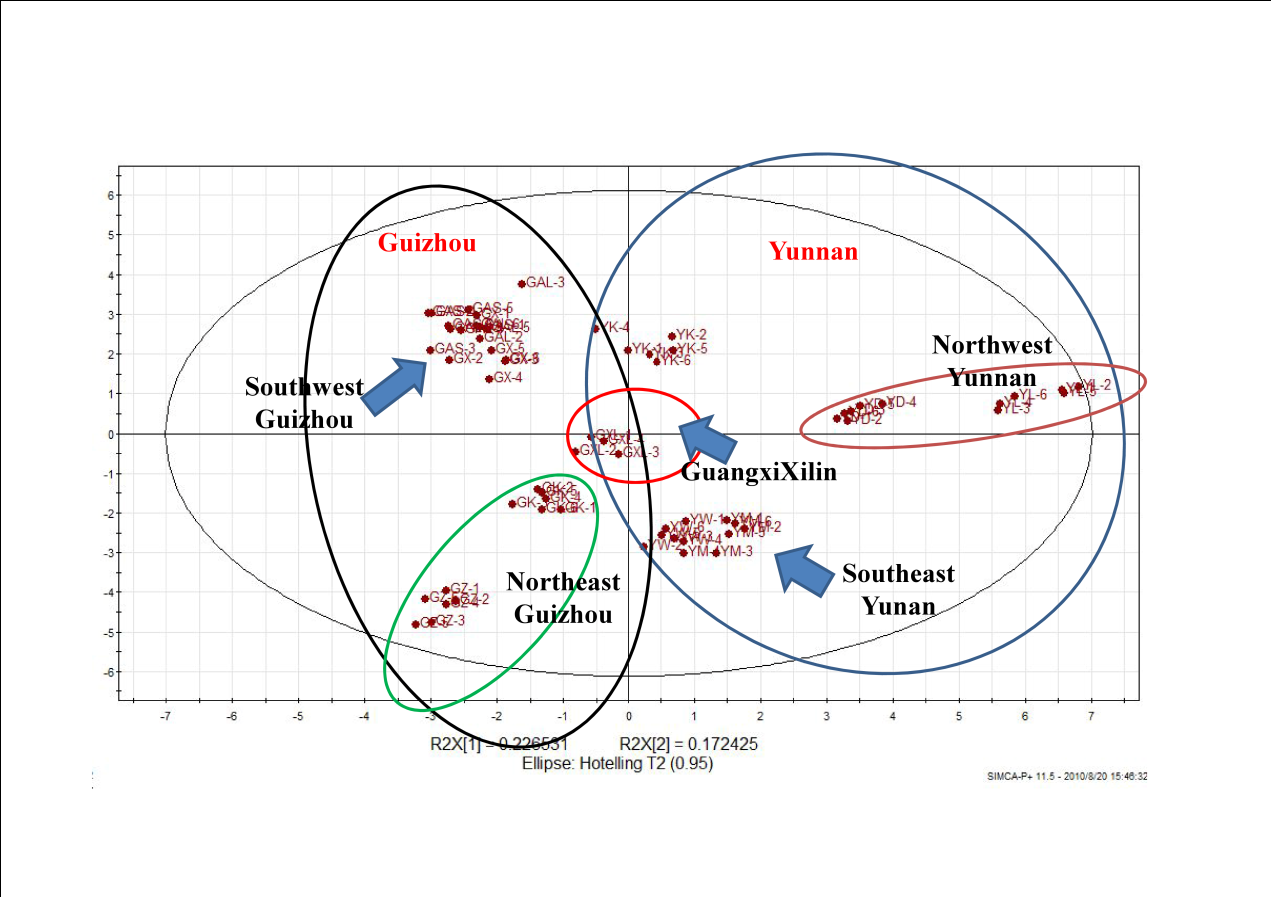

Supplement: Additional file 3: Figure S2. — PCA scores plot on different sites of G. rhodantha. [file 12858_2015_38_MOESM3_ESM.doc]

**Fig. 3S**

**Peak 27**


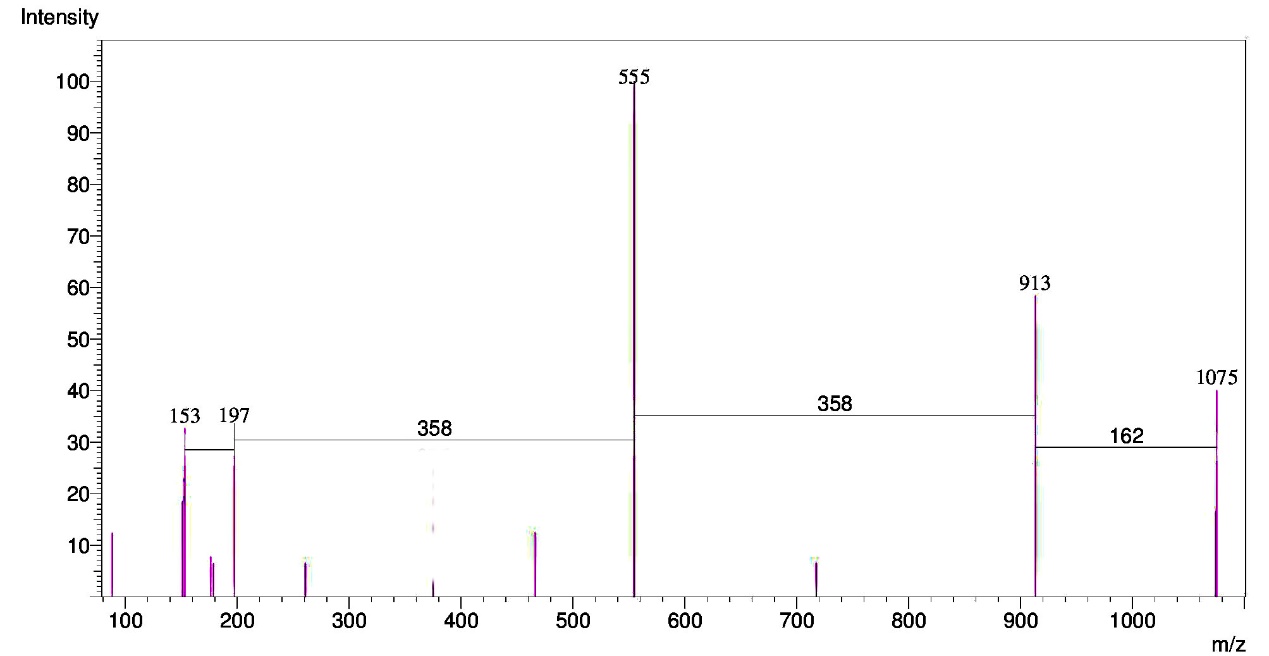


**Peak 28**


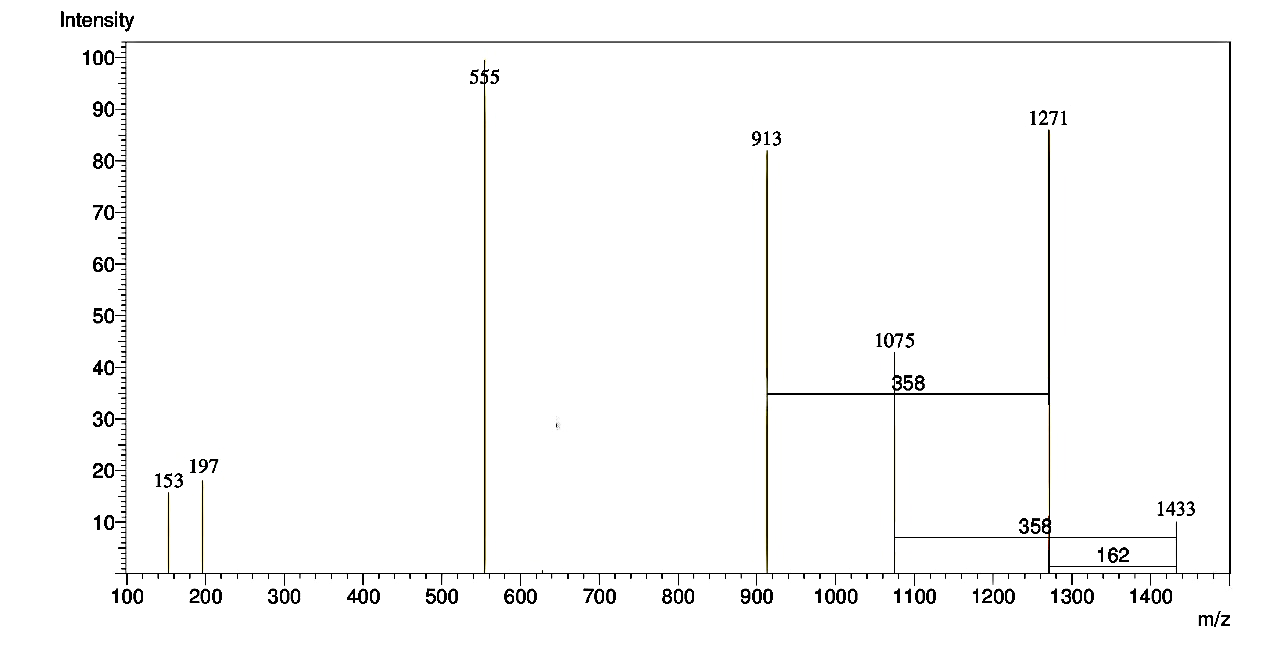


**Peak 31**


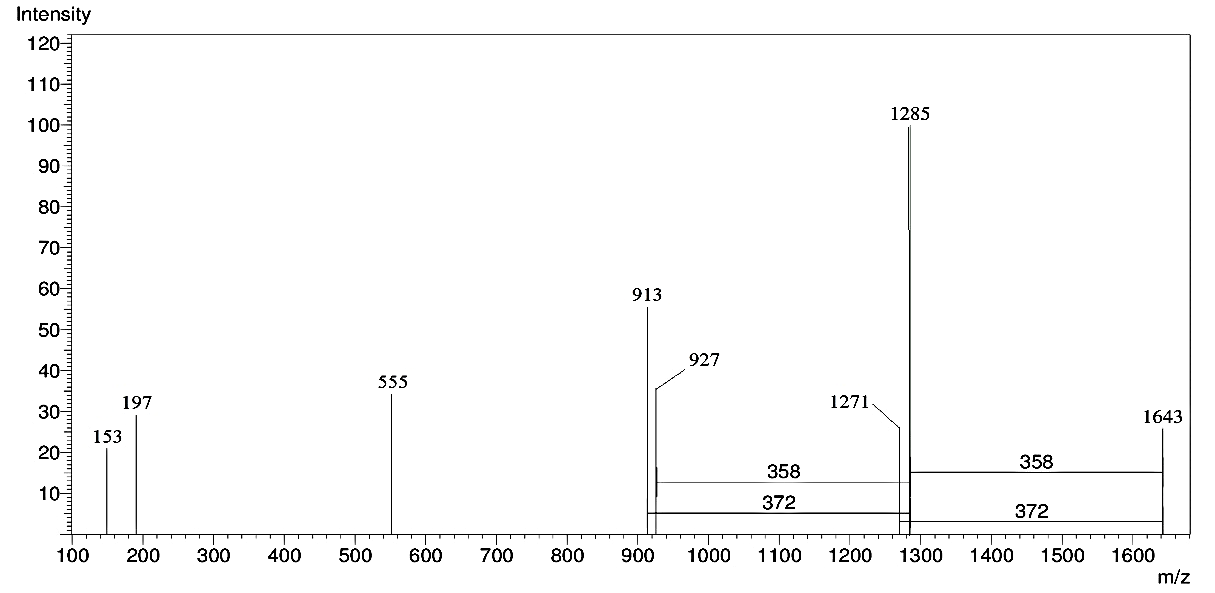

Supplement: Additional file 4: Figure S3. — The MS2 spectrum of the peak 27, 28 and 31. [file 12858_2015_38_MOESM4_ESM.doc]
